# Difficulties in eating out of home while diagnosed with inflammatory bowel disease: A qualitative interview study from China

**DOI:** 10.1371/journal.pone.0288908

**Published:** 2023-12-05

**Authors:** Tingting Yin, Ran Ye, Qiuqin Wang, Lulu Wang, Wenjing Xu, Wenjing Tu, Guihua Xu

**Affiliations:** Nanjing University of Chinese Medicine, Nanjing, China; AIIMS Jodhpur: All India Institute of Medical Sciences - Jodhpur, INDIA

## Abstract

**Background:**

Meeting healthy dietary needs while eating out can be a challenging experience for adults with inflammatory bowel disease. This study examined the barriers experienced by adults with inflammatory bowel disease (IBD) when eating out.

**Objective:**

This study aimed to explore the perceptions of people with IBD on eating out barriers.

**Design:**

A qualitative study among individuals affected by IBD was conducted through semi-structured interviews.

**Results:**

Sixteen adults from China were diagnosed with IBD between 6 months and 20 years prior to the study. They were recruited from four tertiary care hospitals in Nanjing, China. The participants completed a semi-structured interview between April and September 2022. Self-perceived difficulties with eating and drinking when eating out were varied. After thematic analysis of the data, five main themes emerged: limited access to healthy and hygiene food; no pleasure of food enjoyment; financial strain; not feeling loved, supported or understood; and coping strategies for not meeting demand.

**Conclusions:**

This study highlights the various barriers encountered by patients with inflammatory bowel disease when eating out. These findings will help people with IBD to encourage the formation of targeted health and well-being-related interventions. Knowledge of nutrition and diet should be provided in education and training programs administered to IBD.

## Introduction

Inflammatory bowel disease (IBD), which is a group of chronic, relapsing–remitting diseases of the gastrointestinal tract, mainly includes Crohn’s disease (CD) and ulcerative colitis (UC), and its incidence and prevalence are increasing worldwide, especially in developing countries such as China. Diet influences gut inflammation through different mechanisms, including alterations in the composition of gut microbiota and its interactions with the local immune system [1, 2]. Diet, especially the widespread adoption of a Westernized diet, is an important factor in IBD susceptibility and may explain the current epidemiological trends [3]. Palant et al. [4] observed that “diet is often the first behavioral factor manipulated by patients with IBD after symptom onset”. However, current guidelines do not include evidence-based dietary recommendations [5]. Despite the lack of evidence-based guidance on dietary modifications in IBD, many observational studies have described dietary modifications to avoid various foods in patients with IBD, and the burden of food-related behavioral modifications in patients with IBD may produce various non-medical problems, including those related to stigma and co-morbid mental health and psychosocial issues. This condition is especially common in eating out places, which are considered to be a unique feature of social groups [6, 7].

Eating out means eating food prepared by others and consuming it in restaurants, cafes, canteens, and fast-food outlets [8]. In modern societies, urban consumers have become more dependent on highly processed, non-home-prepared foods because of the structural consequences of urbanization that are not conducive to home food preparation. Eating out has become an integral part of daily life and socialization. In a 2019 nationwide survey, 85.8% of participants reported eating out for 1–5 times per week, and 10.1% ate out for more than six times [9]. However, for people with IBD, “eating out” was the most commonly reported impairment in social activities related to food, particularly during active disease, and it is related to gut problems [10]. Zallot et al. [11] noted that one-fifth of the patient group that they studied declined outdoor dining, and a similar percentage does not share the same menu as household members. Considering that no cure is available for this disease, the treatment mainly aims to manage the disease and maximize quality of life [12]. Due to the stressful/discomforting situation caused by gastrointestinal symptoms, some patients may have different eating behaviors, emphasizing their behaviors related to restrictive attitudes. In addition, eating out represents an important part of the contemporary culture of dietary engagement in terms of leisure and social life and work-related meals, a culture that may be significantly influenced by inflammatory bowel disease conditions. However, limited research has been done on the barriers and coping styles of IBD on patients eating out.

Therefore, this study aimed to understand the barriers that adults with IBD encounter when eating out and what coping styles they adopt.

## Materials and methods

### Design

The qualitative study used semi-structured one-to-one interviews and the content was reported in accordance with the Consolidated Criteria for Reporting Qualitative Research (COREQ). This study is based on phenomenology to describe the barriers to eating out in patients with IBD. Interpretivism is an inductive practice influenced by philosophical frameworks such as hermeneutics, phenomenology, and symbolic interactionism. Through this paradigm, we sought to explain the phenomenon of research impact through the individual views and experiences of our interviewees, with the interpretation that these individual experiences also contribute to a broader picture. Therefore, the researcher posed a wide range of questions to the participants, enabling them to interpret the situation independently. All subjects provided informed consent prior to participation in the study. The study was conducted in accordance with the Declaration of Helsinki, approved by the Ethics Committee of Nanjing Chinese Medicine Hospital (KY2022029, approval date 25 February 2022), and completed registration in the Chinese Clinical Trials Registry (ChiCTR2200064943, accessed on 24 October 2022).

### Participants and recruitment

Study participants were recruited through a combination of purposive and convenience sampling. These participants were derived from consecutive outpatients with a confirmed diagnosis of CD or UC who were treated or followed up in the IBD wards of four tertiary general hospitals in Nanjing, China. Participants aged less than 18 years or at least 60 years, and pregnant and lactating women were excluded. Patients were recruited between April and June 2022. Data were collected, and the data are summarized on Microsoft Excel. Demographic information, including age, gender, marital status, education, occupation, place of residence, monthly family income (RMB/person), weekly frequency of eating out (times/week), past and current surgical interventions, medications, type of IBD, and duration of the disease, medication history, and disease activity at the time of examination were recorded. Nutritional status were recorded in terms of current weight, height, body mass index (BMI), presence of food allergies or intolerances, and use of dietary supplements. Disease activity was assessed using the CDAI for CD and the Mayo scale for UC after accurate clinical assessment by an experienced IBD clinician.

### Interview procedure

Interested individuals were invited to send a message to the principal investigator via WeChat. The researcher then confirmed the eligibility of participants and scheduled interviews. All interviews were conducted online via WeChat or over the phone because of the COVID-19 pandemic. The investigator made an appointment with the interviewee at an appropriate time in advance. All interviews were conducted strictly on a one-to-one basis, with no one present except the interviewer and the interviewee. Interviews lasted between 39–152 min, with an average of 70 min. Before every interview, written consent and permission for audio-recording were obtained from participants. Based on the review of literature and research team discussions, an interview guide that included both open-ended and survey-style questions was developed (see S1 File) [13–15]. The reliability of the questions was determined by conducting three pretests prior to the main study, and three of the questions were slightly modified as suggested. However, these three preliminary interviews were not included in the final analysis. Some questions and an interview script were then derived, which represent a study of eating outside the home behaviors that hinder Chinese patients with IBD in locations.

### Data analysis

After all the interviews, the data were analyzed according to the principle of subject analysis. Interviews were summarized in Microsoft Word to facilitate data storage and encoding. The research team followed Colaizzi’s seven-step method with inclusion of an additional step for data analysis, and it includes the following:

1. Transcribing all the subjects’ descriptions;
2. Extracting significant statements, i.e., statements that directly relate to the phenomenon under investigation;
3. Formulating meanings for each significant statement;
4. Organizing formulated meanings into clusters or themes;
5. Integrating results into exhaustive description of the phenomenon;
6. Additional step—interpretative analysis of symbolic representations;
7. Identifying the fundamental structure of the phenomenon;
8. Returning to the participants for validation of the findings.

Interviews were transcribed verbatim into a password protected Microsoft Word document accessible only via a single password protected computer. The coding organization is conducted on this basis using Microsoft Word. No code is predetermined or preset. The team used manual coding to code a word, a sentence, a paragraph, and when they found an example, use a pen to mark the beginning and end of an excerpt and write code in the margin. The preliminary codes were discussed and refined with input from all authors. An operational coding tree was developed and applied to the remaining transcripts by the lead author. The coding tree was developed based on an initial review of the transcripts, and new codes were added to the framework as the two researchers coded individually and conducted regular coding review meetings. A researcher examined the code during all interviews and adjusted and organized it to capture emerging meaning patterns through an iterative process. In this process, the research team organized and refined repeated topics related to potential connections between research questions and topics into an analysis draft, which was formed from sub-topics and topics. The research team constantly reviewed interview transcripts and codes to ensure that the topics reflect the participants’ descriptions of their experiences. All co-authors reviewed and provided feedback on the analysis and interpretation of the results.

### Rigour

The COREQ checklist was followed throughout the study (see S2 File: COREQ Checklist) [14]. The quality and credibility of the results were ensured by adopting various methods. First, researchers began establishing contact with participants in November 2021 to gain patient trust and build rapport. Second, all interviews were conducted by the lead author (T.Y). She is a master candidate who has studied qualitative research knowledge and interview skills systematically and detailedly. T.Y. has prior interviewing and qualitative analysis experience. Finally, throughout the whole process of data analysis, the researchers practiced their reflexivity (that is, reflect on their knowledge and background, and how this may affect the interpretation of the data). More, the whole team refined the theme and content in periodic reviews to ensure consistency.

## Results

**Fig 1:**
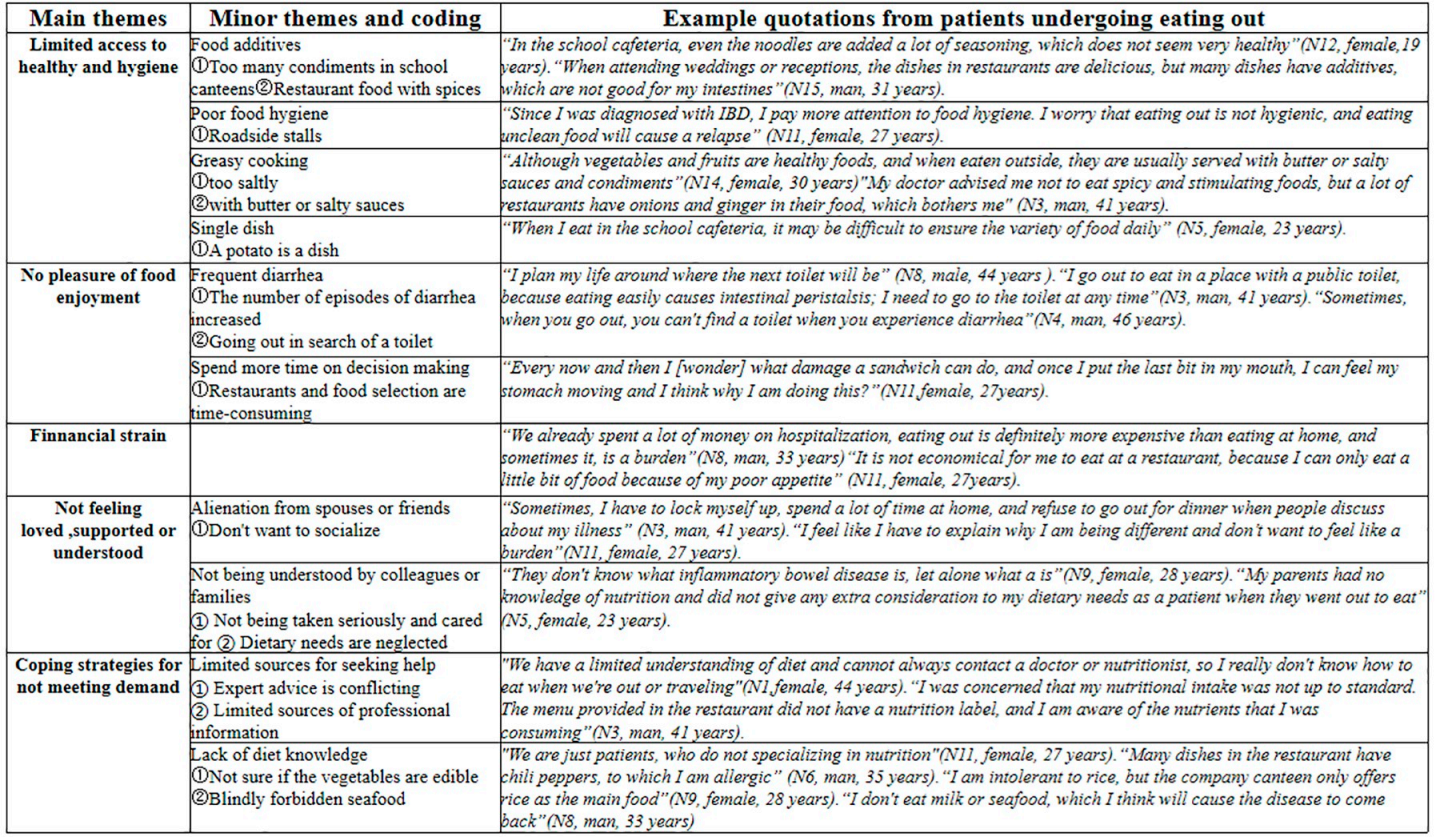
Summary of the thematic analysis of the main theme definitions and sub-theme categories.

We purposively selected participants for interview. And all participants agreed to give an interview after we approached them. Sixteen patients were interviewed. Among the 16 participants, 9 (56.25%) were male, and 7 (42.75%) were female. The average age of participants was 34.81±10.93 years, and the average baseline BMI 21.86±3.07 kg/m^2^ average duration of IBD was 7.12±6.24 years. The characteristics of the included participants are shown in Table 1. Five main themes emerged from the interview data (Fig 1): five main themes emerged: limited access to healthy and hygiene food; no pleasure of food enjoyment; financial strain; not feeling loved, supported or understood; and coping strategies for not meeting demand.

**Table 1:** Summary of patient characteristics of 16 adults with confirmed inflammatory bowel disease who participated in a semi-structured interview about their barriers to eating out after diagnosis.

Characteristics	*n*	(%)	Mean	SD
**Age**			34.81	10.93
**Gender**				
Male	9	56.25%		
female	7	42.75%		
**Marital Status**				
Single	4	25%		
Married	12	75%		
**BMI (kg/m** ^**2**^ **)**			21.86	3.07
**Education**				
Primary and lower	2	12.5%		
Secondary school	5	31.25%		
Bachelor or higher	9	56.25%		
**Occupation**				
Staff	13	81.25%		
Students	1	6.25%		
Unemployed	2	12.5%		
**Region**				
Urban	12	75%		
Rural	4	25%		
**Food allergy or intolerance**				
Yes	6	37.5%		
No	7	43.75%		
Non-reported	3	18.75%		
**Monthly family income (RMB/person)**				
Low (<2,682.42)	5	31.25%		
Middle (2,682.42–5,364.83)	8	50%		
High (>5,364.83)	3	18.75%		
**Weekly frequency of eating out (times/week)**				
1–3	8	50%		
4–6	4	25%		
≥7	4	25%		
**Duration of IBD (years)**			7.12	6.24
**Type of IBD**				
UC	10	62.5%		
CD	6	37.5%		
**Disease Activity**				
Active	6	37.5%		
Remission	10	62.5%		
**Surgery**				
Yes	4	25%		
No	12	75%		
**Medication**				
Yes	15	93.75%		
No	1	6.25%		
**Additional nutritional product**				
Yes	6	37.5%		
No	10	62.5%		

**Fig 2:**
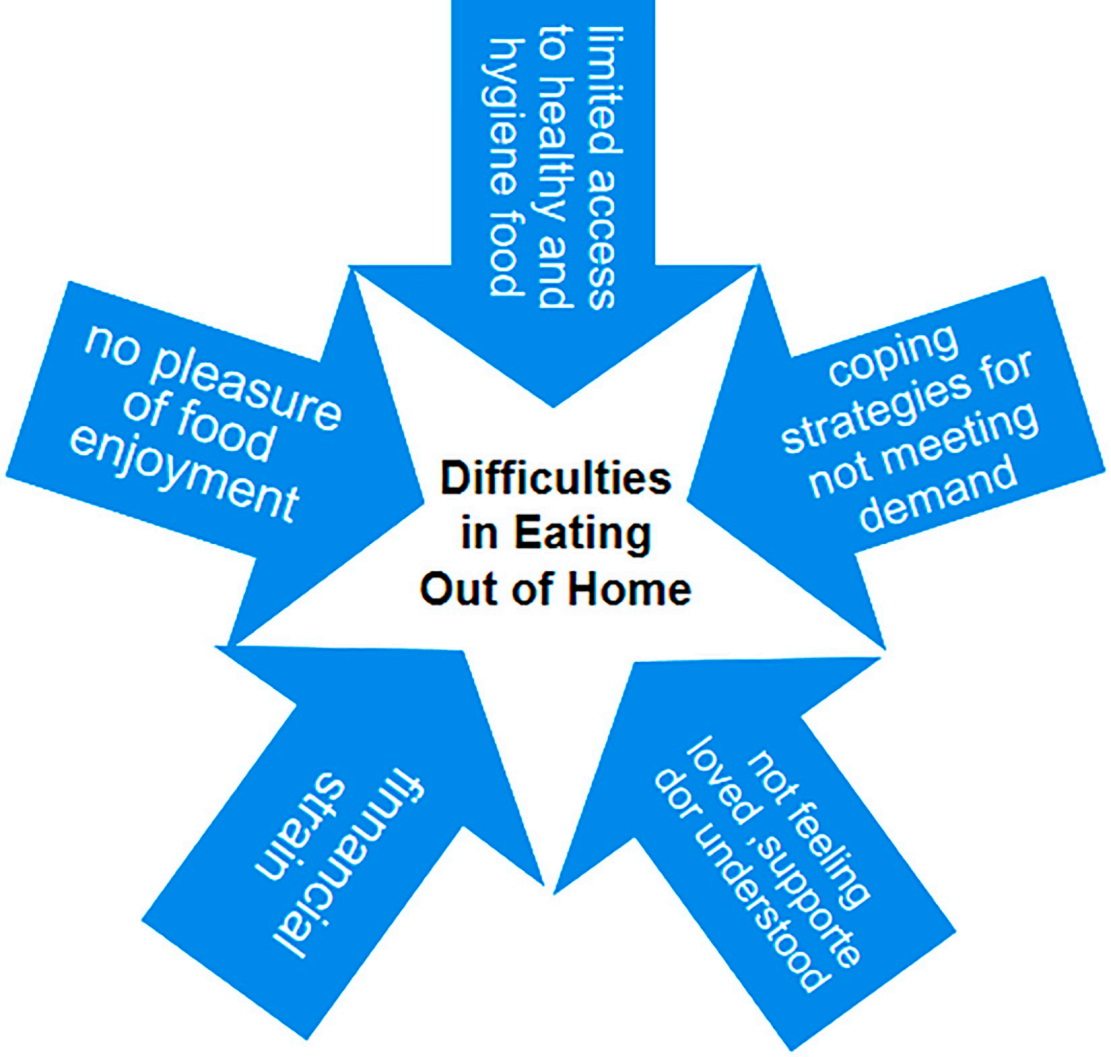
Difficulties in eating out of home while diagnosed with inflammatory bowel disease: A theme framework.

The barriers encountered by patients with inflammatory bowel disease when dining out occur on an individual basis, creating a difficulty that begins with diagnosis and continues throughout the course of the illness. Fig 2 shows the thematic framework throughout the eating out process. The key findings from each of these themes are discussed in the next section.

### Theme 1: Limited access to healthy and hygiene food

Respondents reported a fear of misuse of food additives when eating out, such as in the workplace, restaurants, and other homes, where foods that are unhealthy or contain many additives are likely to be served. “*Although vegetables and fruits are healthy foods*, *and when eaten outside*, *they are usually served with butter or salty sauces and condiments”(N14*, *female*, *30 years)*.*“In the school cafeteria*, *even the noodles are added a lot of seasoning*, *which does not seem very healthy”(N12*, *female*,*19 years)*.*“When attending weddings or receptions*, *the dishes in restaurants are delicious*, *but many dishes have additives*, *which are not good for my intestines”(N15*, *man*, *31 years)*.

Respondents mentioned that the hygiene and safety of some meals, such as self-service kiosks, snacks, fast food, and ready-to-eat food, could not be guaranteed. *“When I ate roadside stinky tofu*, *I started to experience diarrhea after eating” (N7*, *man*, *22 years)*. *“Since I was diagnosed with IBD*, *I pay more attention to food hygiene*. *I worry that eating out is not hygienic*, *and eating unclean food will cause a relapse” (N11*, *female*, *27 years)*. *“I used to work on a construction site and had to eat fast food or roadside food stalls every day*, *which were definitely not clean” (N13*, *man*,*36 years)*.

Respondents described eating out with food that was not served fresh, and then possibly cooked in non-edible oils. *“When I eat at the company on weekdays; the food in the staff canteen does not feel fresh and the quality of raw materials for restaurant food is not guaranteed” (N7*, *man*, *22 years)*. *“Some restaurants use cooking oils that may have been obtained illegally*, *which is unhealthy and may present a risk of contracting infectious diseases or food poisoning” (N2*, *man*, *37 years)*.*“I don’t like eating out*. *A lot of oil and aginomoto are added into the dish*. *I am a patient*, *and the dish at the restaurant is not suitable for me” (N1*, *female*, *44 years)*.

Respondents mentioned that are often one-dish dishes, such as mixed rice, noodles, or dumplings, making it difficult to present a high level of food variety. *“When you eat in the school cafeteria*, *it may be difficult to ensure the variety of food daily” (N5*, *female*, *23 years)*.

### Theme 2: No pleasure of food enjoyment

Respondents indicated that they were aware about having gastrointestinal problems, which require them to go to the restroom more often than others. *“I go out to eat in a place with a public toilet*, *because eating easily causes intestinal peristalsis; I need to go to the toilet at any time”(N3*, *man*, *41 years)*.*“Sometimes*, *you have to go to the toilet for several times; I used to go for five times*, *but now it’s 10*, *especially when you’re not in remission” (N15*, *man*, *31 years)*.

The concern among participants that eating anything requires bowel movement has also been supported during a flare. *“I don’t eat breakfast when going out*, *because I know that within a couple of minutes of eating*, *I will need to go to the bathroom” (N3*, *man*, *41 years)*. *“When I’ve got a flareup going*, *I need to go to the bathroom half an hour after eating*.*” “I am embarrassed when I eat out with others” (N14*, *female*, *30 years)*.

For many participants, IBD meant that dining out or travelling needs to be planned around the location of the restroom. *“I plan my life around where the next toilet will be” (N8*, *male*, *44 years)*. *“When I was a guest at a friend’s house*, *I suddenly lost control of my intestines and didn’t have time to go to the toilet and stained my underwear and pants”(N1*, *female*, *44 years)*.*“I hope there is no line*, *what if there is*, *what if I can’t wait*? *The fear is there*. *What if I make a mess*? *Fear of not going to the bathroom affects your quality of life the most” (N11*, *female*, *27 years)*.

Respondents reported the need for more extensive meal planning when eating out and often spend more time in choosing restaurants and limited food. *“Every now and then I [wonder] what damage a sandwich can do*, *and once I put the last bit in my mouth*, *I can feel my stomach moving and I think why I am doing this*?*”(N11*,*female*, *27years)*. Participants acknowledged a large amount of anxiety resulting from a pattern of their symptoms controlling their lives and the resulting effects of their disease on their quality of life. *“You end up planning your whole life around what your gut is doing” (N8*, *man*, *44 years)*. *“When it started*, *I just had to stay home*, *because I often couldn’t handle it and had absolutely no control”(N16*,*female*,*49 years)*.

### Theme 3: Financial strain

Respondents report more financial burden on families because of the cost of eating out.*“We already spent a lot of money on hospitalization*, *eating out is definitely more expensive than eating at home*, *and sometimes it*, *is a burden”(N8*, *man*, *33 years)*.*“Eating at the restaurant is too expensive for me to afford*. *I have been sick for 12 years*, *and the disease is a heavy economic burden for my family” (N3*, *man*, *41 years)*. *“Most people eat out*, *and the quality of the diet should be determined by economic conditions; many people are financially incapacitated”(N16*, *female*,*49 years)*. Even a patient reviewed eating out of home as wasting because of loss of appetite. *“It is not economical for me to eat at a restaurant*, *because I can only eat a little bit of food because of my poor appetite” (N11*, *female*, *27years)*.

Respondents described having to plan and buy food on a lower budget when eating out, which requires buying cheaper food with less nutritional value. *“I often order at McDonald’s but usually only order a set menu and then bring it over to eat”(N3*, *man*, *41 years)*.*”I wanted to buy fresh fruits*, *vegetables*, *and protein-rich foods*, *but they are expensive*, *so I had to give them up”(N16*, *female*, *49 years)*.*”When I have to eat somewhere other than home*, *I pick a restaurant that offers a discount or a deal”(N8*, *male*, *44 years)*. Limited and unstable income may limit an individual’s ability to access nutritious foods and maintain healthy eating habits. “*After developing an illness*, *I often missed work*, *and my income was unstable*, *so eating out at low prices was my primary consideration”(N9*, *female*, *28 years)*.

### Theme 4: Not feeling loved, supported or understood

Respondents mentioned losing touch with food after illness, lack of understanding of illness and nutrition by friends, and often being isolated when eating out. *“They don’t know what inflammatory bowel disease is*, *let alone what a Full Liquid Diet is”(N9*, *female*, *28 years)*. When eating out, friends or restaurants are not aware of the patient’s history of special dietary needs, such as food allergies and food intolerances.*“My parents had no knowledge of nutrition and did not give any extra consideration to my dietary needs as a patient when they went out to eat”(N5*, *female*, *23 years)*. Respondents are often reluctant to discuss their illness with friends and relatives but cannot avoid it when dining out.*“Sometimes*, *I have to lock myself up*, *spend a lot of time at home*, *and refuse to go out for dinner when people discuss about my illness”(N3*, *man*, *41 years)*.*“I don’t want to affect others’ decisions about where they can eat”(N5*, *female*, *23 years)*.*“I feel like I have to explain why I am being different and don’t want to feel like a burden”(N11*, *female*, *27 years)*. Some respondents did not want to be a burden to their friends or family to ask for help when dining out because of the stigma of illness. *"When I eat out*, *I don’t prefer getting a special treatment" (N16*, *female*, *49 years)*.

### Theme 5: Coping strategies for not meeting demand

Respondents reported a range of strategies to overcome these barriers when eating out, such as reading food labels as a way to adjust recipes, *“When I go out to eat*, *I make reservations in advance to look for a suitable menu and food and try to choose something to eat”(N4*, *man*, *46 years)*, finding "safe" restaurants *“If I have to eat out*, *I choose restaurants that clean and light in taste*”*(N5*, *female*, *23 years)*, trying new foods *“When we get together with colleagues*, *I try new food so as not to cause trouble for others*”*(N16*, *female*,*49 years)*, bringing food to other people’s homes and workplaces *“I will prepare food at home before I leave home in the morning*, *pack it*, *take it to the office*, *and heat it up in the microwave when I eat at noon*” *(N2*,*man*,*37years)*, and planning meals and trips out in advance *“You need to be prepared before going out” (N14*, *female*,*30 years)*; *“I have to make sure that we do anything in the morning or that I’m going anywhere the next day*…… *I have to make sure I get up in time to go three*, *four*, *or five times before I go to the bathroom” (N15*, *man*, *31 years)*.

Many respondents described several strategies to address barriers to eating out. Almost all respondents described limited sources for seeking help and the lack of up-to-date knowledge about managing eating out with IBD. *"We have a limited understanding of diet and cannot always contact a doctor or nutritionist*, *so I really don’t know how to eat when we’re out or traveling"(N1*, *female*, *44 years)*.*"We are just patients*, *who do not specializing in nutrition"(N11*, *female*, *27 years)*. *“Sometimes*, *when you go out*, *you can’t find a toilet when you experience diarrhea” (N4*, *man*, *46 years)*. Respondents complained about inadequate nutrition labeling and allergen information on menus. *“I was concerned that my nutritional intake was not up to standard*. *The menu provided in the restaurant did not have a nutrition label*, *and I am aware of the nutrients that I was consuming”(N3*, *man*, *41 years)*.*“I am intolerant or allergic to many food ingredients*, *but the ingredients are not provided in the menu every time I eat out”(N4*, *man*, *46 years)*.*“When you go to a restaurant*, *you are unaware of the ingredients in the food*, *and you don’t know if you’re eating well at all”(N8*, *man*, *44 years)*. Patients stated that not knowing the type of food to eat is a barrier when they have to choose what to eat outside home. *“Many dishes in the restaurant have chili peppers*, *to which I am allergic” (N6*, *man*, *35 years)*.*“I am intolerant to rice*, *but the company canteen only offers rice as the main food”(N9*, *female*, *28 years)*.

## Discussion

Qualitative approach was used to examine how eating out affects patients with IBD in China. Data generated five themes, namely, limited access to healthy and hygiene food; no pleasure of food enjoyment; financial strain; not feeling loved, supported or understood; and coping strategies for not meeting demand. These themes demonstrate that the factors influencing dietary intake are complex and multi-faceted. It provides new insights into this area and provide practical information on barriers to eating out.

Lack of healthy and hygiene food is the main barrier to eating out. Foods provided by out-of-home food establishments may be a characteristic component of unhealthy eating patterns. The majority of participants felt somewhat insecure about their exposure to processed and low-quality foods prevalent in eating out, as well as social practices and norms around snacking. Participants perceived that the inclusion of excessive oil and additives in foods eaten away from home and restaurant foods, and foods high in fat and sugar increase the intake of restrictive nutrients, and this dietary pattern could negatively affect gut health. In addition, eating out is associated with higher energy and fat intake and lower micronutrient intake [16], which supports the participants’ concerns about the health effects of eating out. The results of the present study show that food hygiene or cleanliness is important for people with IBD. Participants reported that food prepared outside the home is often unclean. Eating out may be associated with improper food preparation, inadequate refrigeration temperatures, and poor personal hygiene of food service staff. Several factors determine how consumers choose food establishments [8, 17, 18]. These factors include food quality, hygiene and food safety, taste, cleanliness, staff behavior, location, reputation, and price. Participants also reported that many roadside stands or street foods do not pay enough attention to food hygiene during food preparation and handling, which greatly increases the risk of food safety and that unhygienic food may aggravate or trigger the condition of patients with IBD. In addition, more fresh, tasty, and high-quality meals should be provided. Recommendations included reducing salt, flavor enhancers, and providing more fresh and appealing vegetables. Offering a wider variety of healthy meals, especially clean meals, was recommended.

No pleasure of food enjoyment, particularly toilet accessibility and time costs, were mentioned by participants in interviews. Similar to other studies, people with IBD reported physical symptoms, such as diarrhea and abdominal pain, which may deteriorate their diet outside home [19]. Most of the participants mentioned that eating out centered on food safety leads to limited enjoyment and conviviality. Spontaneous and unplanned eating is no longer practiced because of the focus on access to safe food. Some participants also reported frequent uncontrollable diarrhea during periods of active illness when traveling, visiting friends and family, or traveling for business, thus preventing them from enjoying meals away from home. In China, uncontrolled bowel movements are strictly prohibited, because they are considered a private behavior. Therefore, many participants said that they were afraid to eat outdoors, because they often lose control of their bowel movements and could not find a toilet or hiding place in time in unfamiliar surroundings. Diarrhea is embarrassing in terms of the trouble of finding a toilet and can also be life-threatening in severe cases because of dehydration. In addition to uncontrollable bowel symptoms every time they eat out, people with IBD have various non-medical problems, including stigma-related issues and comorbid mental health and psychosocial problems [19, 20]. In addition, when going out to choose a restaurant, the act of eating out requires more effort required by dietary restrictions of people with IBD, because the preparation of safe food requires time and effort. Each participant described the burden of going out to plan a shopping list, deciding which restaurants to eat at, and then the extra time required to read the label once at the restaurant.

The participants also expressed concerns about the economy. Patients with IBD have higher rates of hospitalization and prescription drug use, placing a greater financial burden on them. Affordability is important to participants, because patients with chronic conditions have limited budget, as expected. Financial insecurity affects patients’ spending levels, purchasing power, and eating habits outside their home. Studies from other countries report that most menu developers consider increasing sales and profits as the main consideration when offering menus. Notably, people with inflammatory bowel disease consider health and nutrition to be important aspects of their menus. In addition, street food offers foods made from inexpensive ingredients, and this type of diet is very popular among low-income patients. Participants expressed that outside foods are more expensive than home-made ones, thus creating a barrier for patients with IBD in adjusting their eating behaviors when eating out. A systematic evaluation showed that eating out is associated with higher socioeconomic status, which was consistent with participants’ concerns about cost [21]. In the present study, financial pressures forced some participants to choose cheaper foods over healthier ones. Low socioeconomic status influences the quality of the out-of-home diet, in which lower-income groups consume more sweet because of the low cost of purchasing "unhealthy" foods outside the home [9]. Several studies have looked at the food environment of college students, offering discounts on fruit purchases or taxes on sugary drinks, and have shown an increase in healthy food purchases [22, 23]. Therefore, price reduction should be considered as an option for patients with IBD to improve the nutritional value of foods consumed outside home. Moreover, access to quality food should be improved through the establishment of institutional food banks or affordable cafeterias.

The social dimension of the restrictive eating out experience changed profoundly [24]. Participants described a loss of connection and enjoyment around food considering that eating became more monolithic or food-centered for safety and their IBD. Individuals described the isolating nature of having to bring their own food or ask for separate food at family dinners, restaurants, and the decreased ability to travel. Individuals described how social situations caused feelings of being a burden, embarrassment, or inconvenience to others. Family rituals and traditions led to further isolation and stress, considering that some individuals had to bring their own food, had to ask how the food was prepared, and lacked the trust and concern about how others prepare the food. In addition, individuals described feelings of worry, sadness, and/or grief when eating out, losses caused by closing food-related businesses, loss of friendships, or changes in the dynamics of family rituals after illness. The study confirmed that many consumers are looking for more information when eating out and want to consider the composition and nature of the food they consume. Barley and wheat has become a common source of energy and nutrient intake in Chinese diet and are the most popular foods served and eaten outdoors; however, for people with IBD who may have gluten intolerance, a gluten-free diet is required, indicating the need for completely gluten-free products such as gluten-free bread, pizza, some fast foods, and other staples [25, 26]. The management of food allergies and intolerances relies on avoiding the food components and products that cause them; therefore, affected individuals must adopt a lifestyle of constant vigilance. To avoid accidental ingestion, many countries have implemented allergen labeling regulations. Proper food labeling helps consumers with food allergies or intolerances identify the products that they should avoid and make safe choices. Accordingly, the Chinese government should be required to use clear and source labeling for ingredients from common allergens.

Limited social support is among the external factors that contribute to barriers to eating out. Participants described the fear, worry, and trust of food security as a person’s arena. Dietary changes caused by disease states may lead to difficulties in reassessing identity with understanding and support from family or close friends. The restrictive nature of the diet and social isolation may lead to a loss of connection to and enjoyment of food, and this feeling may cause resentment and amplification of the illness at the expense of quality of life [12, 21, 27]. Notably, individuals with IBD can be fully engaged in all dimensions of the eating experience prior to diagnosis, but after diagnosis, individuals may experience a loss of identity associated with multiple dimensions of the eating experience [28]. The lack of social support weakens participants’ social connections, creating a sense of socially alienated isolation and depriving them of material or emotional dependence and avenues to relieve stress and tension; this condition may result in a loss of anxiety about their illness, associated with eating out, or eating spontaneity. This loss may have deteriorate physical and/or psychosocial well-being.

Participants mentioned strategies that can address barriers to eating out through various factors, such as personal improvement and opportunities to go out to places. First, most participants agreed that lifestyle changes, such as adapting recipes to find food alternatives that work for them, are essential to promote and enhance personal improvement in eating out. In addition, participants agreed that finding "safe" restaurants, reading labels, and trying new foods can promote eating out. In addition, participants could bring food to other people’s homes and workplaces, and plan meals and trips out in advance so that they can eat outside at work if necessary. However, while many respondents described several strategies to address barriers to eating out, almost all participants described limited sources for seeking help and a lack of up-to-date knowledge about managing eating out with IBD.

This study had some limitations with respect to the sample. Although a sufficient number of subjects was recruited to achieve data adequacy, the patients included in the study were from the same country, which casts doubt on the generalizability of the results to similar groups. These results are not statistically representative and merely represent a phenomenological explanation to help understand the factors that impair the perception of people with IBD when eating out.

## Conclusions

In conclusion, patients with IBD are very concerned about their personal behavior when eating out and the nutritional value of the foods they choose. Qualitative methods provide an opportunity to understand the factors and actual components of barriers to eating out after a diagnosis of IBD from the patient’s perspective. Our findings highlight the complex set of barriers experienced by patients with inflammatory bowel disease when eating out. These barriers include limited access to healthy and safe food supplies and financial constraints, loss of ease of living, lack of access to social support and coping skills, and internalization of negative emotions. From a clinical perspective, recognizing the dimensions of the barriers experienced by people with IBD when dining out in the context of nutrition practice can help inform the development of interventions to effectively maintain the quality of life and psychological well-being of patients associated with dining out.

## Supporting information

S1 File Interview topic guide. (DOCX)

S2 File Consolidated criteria for reporting qualitative studies (COREQ): 32-item checklist. (DOCX)
