# Supplementary material for: Difficulties in eating out of home while diagnosed with inflammatory bowel disease: A qualitative interview study from China
Source: PLoS One. 2023 Dec 5;18(12):e0288908. doi: 10.1371/journal.pone.0288908 (PMC10697536; doi:10.1371/journal.pone.0288908)
Supplement: S1 File — (DOCX) [file pone.0288908.s001.docx]

**Supporting Information 1**

**Interview topic guide**

**Introduction**

Interviewer introduces themselves, and the aims of the study. Ground rules:

- Participant is free to state at any time if they feel uncomfortable with questions or want to stop the interview.
- The interview will be audio recorded and the interviewer will make brief notes. Both will be anonymised after the interview.
- There are no right or wrong answers and all responses are valid.

**Prompts**

- First, I want to learn more about your current diet. Please tell me how your daily diet typically looks like?How often do you eat out?
- Tell me about the meals that you’ve had over the past week. Did you eating out? What did you prepare?

**General questions**

- How do you personally define eating out?
- What role does eating out play for you?
- To what extent would you say you follow a normal diet when eating out?
- What does eating out mean to you?
- How do you feel about eating out (eg enjoyable, a nuisance)?Why?
- Are there particular aspects that you do or don’t like? Why?

**Determinants**

- Where do you eat your meals? Why?
- Do you eat with anyone else? Who and why?
- What factors make it difficult for you to eating out? (eg too busy, ingredients are expensive, don’t have necessary skills)
- What factors would you like to change?
- Who has an impact on what and how you eating out?
- Does the healthiness of foods influence your eating out patterns?
- What comes before health in your priorities and why?

**Wrap up**

- Is there anything else you’d like to mention that we haven’t covered?
- Close; thank the participant; and provide debriefing sheet.
