# Supplementary material for: Difficulties in eating out of home while diagnosed with inflammatory bowel disease: A qualitative interview study from China
Source: PLoS One. 2023 Dec 5;18(12):e0288908. doi: 10.1371/journal.pone.0288908 (PMC10697536; doi:10.1371/journal.pone.0288908)
Supplement: S2 File — (DOCX) [file pone.0288908.s002.docx]

**Supporting Information 2**

Consolidated criteria for reporting qualitative studies (COREQ): 32-item checklist

| **No** | **Item** | **Guide questions/description** |
| --- | --- | --- |
| **Domain 1: Research team and reflexivity** | | |
| Personal Characteristics | | |
| 1. | Interviewer/facilitator | TY |
| 2. | Credentials | TY(MSc), RN (PhD), QW (PhD), LW (MSc), WX(MSc),WT(PhD),GX(PhD) |
| 3. | Occupation | TY(Student),RN(Professor of Nursing), QW(Nutrition Specialist), LW (Student), WX(Student),WT (Inflammatory bowel disease and Research Specialist), GX (Professor of Nursing) |
| 4. | Gender | female |
| 5. | Experience and training | The team members have all received rigorous study and training in qualitative research, and TY has some experience with qualitative interviews. |
| Relationship with participants | | |
| 6. | Relationship established | Yes. |
| 7. | Participant knowledge of the interviewer | Few participants were known to the YT. |
| 8. | Interviewer characteristics | All interviewers are public nutrition and inflammatory bowel disease researchers and are particularly interested in qualitative methods. |
| **Domain 2: Study design** | | |
| Theoretical framework | | |
| 9. | Methodological orientation and Theory | Qualitative methods and thematic analysis, phenomenology |
| Participant selection | | |
| 10. | Sampling | Purposive and convenience sampling |
| 11. | Method of approach | For all interviews were conducted online via Wechat or over the phone. |
| 12. | Sample size | 16 |
| 13. | Non-participation | Zero.We purposively selected participants for interview. And all participants agreed to give an interview after we approached them. |
| Setting | | |
| 14. | Setting of data collection | Word Office |
| 15. | Presence of non-participants | No |
| 16. | Description of sample | See Table 1 |
| Data collection | | |
| 17. | Interview guide | An interview guide was drafted, piloted，see supporting information 1 |
| 18. | Repeat interviews | None |
| 19. | Audio/visual recording | All interviews were audio-recorder. |
| 20. | Field notes | Yes |
| 21. | Duration | 39–152 minutes |
| 22. | Data saturation | Yes |
| 23. | Transcripts returned | Yes |
| **Domain 3: Analysis and findings** | | |
| Data analysis | | |
| 24. | Number of data coders | No |
| 25. | Description of the coding tree | See Figure 1 |
| 26. | Derivation of themes | Theme were derived from the data based on interpretivism approach |
| 27. | Software | No |
| 28. | Participant checking | Yes |
| Reporting | | |
| 29. | Quotations presented | Yes |
| 30. | Data and findings consistent | Several relevant quotations used to illustrate findings. |
| 31. | Clarity of major themes | Yes |
| 32. | Clarity of minor themes | Yes |
